# Photobiomodulation in Medication-Related Osteonecrosis of the Jaw: Outcomes in Stage I and Its Adjunctive Role in Advanced Cases

**DOI:** 10.3390/biomedicines13051042

**Published:** 2025-04-25

**Authors:** Filip Michalak, Marzena Dominiak, Zuzanna Grzech-Leśniak, Jan Kiryk, Kinga Grzech-Leśniak

**Affiliations:** 1 Dental Surgery Department, Wroclaw Medical University, 50-367 Wroclaw, Poland; filip.michalak@umw.edu.pl (F.M.); marzena.dominiak@umw.edu.pl (M.D.); jan.kiryk@umw.edu.pl (J.K.); 2 Faculty of Medicine and Dentistry, Wroclaw Medical University, 50-367 Wroclaw, Poland; zuzanna.grzech-lesniak@student.umw.edu.pl; 3 Department of Periodontics, School of Dentistry, Virginia Commonwealth University VCU, Richmond, VA 23298, USA

**Keywords:** osteonecrosis of the jaws, MRONJ, prevention, photobiomodulation, PBM, laser

## Abstract

**Background/Objectives**: The development of pharmacotherapy, particularly in antiangiogenic drugs, has led to the emergence of MRONJ as a significant side effect. With the increasing incidence of cancer, the management of MRONJ poses a growing challenge for clinicians. The aim of the study is to evaluate the effectiveness of photobiomodulation (PBM) in treating patients with stage I, II, and III medication-related osteonecrosis of the jaw (MRONJ). **Methods**: A total of 31 patients were divided into two groups: Group 1 (n = 14 patients), with Stage 1 MRONJ; and Group 2 (n = 17 patients), with Stage II and III MRONJ. In total, 10 patients had osteoporosis and 21 underwent cancer treatment. The sole variable under investigation was the stage of MRONJ, as all patients underwent the same photobiomodulation (PBM) procedure. For treatment protocol, PBM with a diode laser was used (Lasotronix Smart M Pro, Piaseczno, Poland) with the following parameters: 100 mW; continuous wave; 635 nm; 4 J/cm^2^ for 20 s; irradiance for one point: 0.398 W/cm^2^; fluency for one point: 7.96 J/cm^2^, and for four points, which was one appointment: 31.83 J/cm^2^; and tip diameter 8 mm (three points from buccal surface, perpendicular for the lesion and one point on the floor of the mouth) during each session. The protocol assumed 10 sessions at 3 days intervals. Antibiotic therapy (amoxicillin with clavulanic acid 875 mg + 125 mg or clindamycin 600 mg every 12 h) was started 3 days before PBM and continued for 14 days. Antibiotics were taken for 14 days in total. Pain was measured with VAS scale. Follow-up was after 3 and 6 months. **Results**: Among the 14 patients in Group 1, none exhibited any clinical signs or symptoms of MRONJ during the 3 months follow-up, and complete cure was achieved. While PBM resolved inflammation and pain in stage II MRONJ, further surgical intervention was necessary to fully address the condition. **Conclusions**: PBM is an effective treatment for achieving complete recovery in patients with Stage 1 MRONJ. However, in Stages II and III MRONJ, PBM significantly alleviates symptoms but requires complementary surgical intervention to achieve full resolution. A beneficial aspect is the reduction in pain symptoms and the extent of surgical intervention.

## 1. Introduction

While advances in pharmacology have significantly improved the treatment of various medical conditions, they have also introduced potential risks associated with certain therapies. Patients with skeletal system disorders, such as osteoporosis [1,2,3] and Paget’s disease [4,5], as well as patients with specific cancers, including breast [6,7] and prostate [8,9] cancer, are frequently prescribed bisphosphonates and RANKL (Receptor Activator of Nuclear Factor Kappa-B Ligand) inhibitors. However, the use of these medications has been associated with adverse effects, most notably the development of osteonecrosis of the jaw (ONJ) [10]. Medication-related osteonecrosis of the jaw (MRONJ) is defined by specific criteria: a current or previous history of antiresorptive or antiangiogenic drug use, exposed bone that can be probed through an intraoral or extraoral fistula in the orofacial region, persistence of the condition for at least 8 weeks, and no prior history of radiotherapy to the jaw [11,12].

The first case of osteonecrosis of the jaw was described in 2003 by Marx et al. [13]. Pamidronate (Aredia) and zoledronate (Zometa) potent nitrogen-containing bisphosphonates, that are difficult to metabolize accumulate in bone tissue, have been associated with the development of necrotic lesions [14,15,16]. A few years later, it was also discovered that denosumab, another drug acting on the skeletal system, may cause similar complications. The pathogenesis of MRONJ has been extensively studied and is thought to be linked to the inhibition of bone remodeling through osteoclasts suppression, which may lead to bone sclerosis and ischemia [17,18,19]. MRONJ is a disease with a complex etiopathogenesis, and despite many years of research, it is still not possible to identify a single, definitive cause of its development.

MRONJ is a disorder of complex etiology. A detailed medical history is a fundamental to the diagnosis and management of this condition. One of the primary risk factors directly associated with its development is the use of bisphosphates, which have a destructive effect on bone metabolism [19]. Research shows that patients receiving these medications intravenously are at a significantly higher risk of developing the disease compared to patients taking them orally [20]. The treatment pattern for certain cancers, such as breast and prostate cancer, as well as multiple myeloma, commonly includes bisphosphonate therapy, thereby substantially increasing the risk of MRONJ in these patient populations [21,22,23]. Recent studies indicate that MRONJ most frequently develops following surgical interventions in the oral cavity among high-risk patients [24]. The most common procedures include tooth extractions, which, if not properly planned, or if the patient is not adequately prepared, may lead to the development of jawbone necrosis [25,26]. In MRONJ, primary prevention plays a very crucial role. Proper preoperative planning, including antibiotic prophylaxis and assessment of vitamin D levels, can greatly reduce the risk of postsurgical complications and the onset of MRONJ [27]. In the case of bisphosphonates, the time of administration is also an important factor. Studies have shown that patients treated with bisphosphonates for more than 4 years are at greater chance of developing MRONJ compared to those with a shorter treatment duration. In addition to bisphosphonates, denosumab, which is often used in osteoporosis treatment, is also considered a significant risk factor. According to research from 2020, denosumab may have an even stronger association with MRONJ development than zoledronic acid (a bisphosphonate, BP) [28].

With the advancement of laser dentistry and the development of new treatment protocols, both low- and high-power lasers are now effectively used in the treatment of MRONJ [29,30]. In addition to conventional surgery, Er:YAG lasers have been used in the treatment of extensive necrotic lesions in the maxillary and mandibular bones. Low-power lasers, on the other hand, have demonstrated a positive effect on the healing process and are particularly helpful in managing localized and less advanced lesions, allowing clinicians to avoid trauma associated with standard surgical procedures [31].

In modern dentistry, laser technology has become increasingly common, due to its precision and minimal invasiveness [32]. The laser beam is very well absorbed by water, hemoglobin, and melanin, which allows for biologic activity within the tissues [33]. When applied at higher power levels, the laser can have a destructive effect, making it suitable for surgical procedures. The benefits of laser application allow it to minimize the risk of infection, decrease postoperative pain and swelling, improve healing, and provide a high level of precision through targeted energy delivery [34,35,36]. In patients with MRONJ where bone metabolism is impaired, laser-assisted treatment can support successful healing. Photobiomodulation (PBM) utilizes red or infrared light to act at the cellular level [37]. It enhances the production of adenosine triphosphate (ATP) and triggers the release of specific levels of reactive oxygen species (ROS) and nitric oxide (NO). The release of NO promotes vasodilation, improves microcirculation, and accelerates tissue repair processes [38,39,40]. The therapeutic wavelength for PBM typically falls within the range of 600–1200 nm [41]. In the treatment of MRONJ, lasers employing photobiostimulation can serve multiple roles. A study by Vescovi et al., involving over 500 patients, demonstrated that PBM used after the surgical treatment may serve as a preventive function in high-risk patients (e.g., those with cancer or osteoporosis), significantly reducing the likelihood of MRONJ development [42]. Additionally, photobiomodulation provides analgesic and anti-inflammatory effects, and in certain cases, may be applied palliatively when surgical intervention is contraindicated or not feasible [43,44,45].

The study aimed to evaluate the effectiveness of photobiomodulation therapy (PBM) in the treatment of medication-related osteonecrosis of the jaw (MRONJ) using a 635 nm diode laser in different Stages of MRONJ. Specifically, the study sought to assess the following objectives: (1) how PBM relieved pain and inflammation while promoting recovery from necrosis; (2) the effectiveness of PBM in improving clinical outcomes among patients with varying severity levels of MRONJ; (3) the relationship between the degree of necrosis prior to treatment and the subsequent healing process; and (4) whether PBM has long-term effects on pain intensity over a 6-month observation period.

## 2. Materials and Methods

The clinical study was carried out in the Department of Oral Surgery at Wroclaw Medical University, Poland. A total of 31 patients were selected for the diagnosis and treatment of MRONJ. Patients were supported throughout every stage of the process, including diagnosis, treatment, and follow-up at 3 and 6 months after therapy initiation. The study protocol and treatment regimens were approved by the Bioethics Committee (No. KB-27/2021). The primary objective of this study was to evaluate the effects of 635 nm diode wavelengths and their role in managing MRONJ.

**Figure 1:**
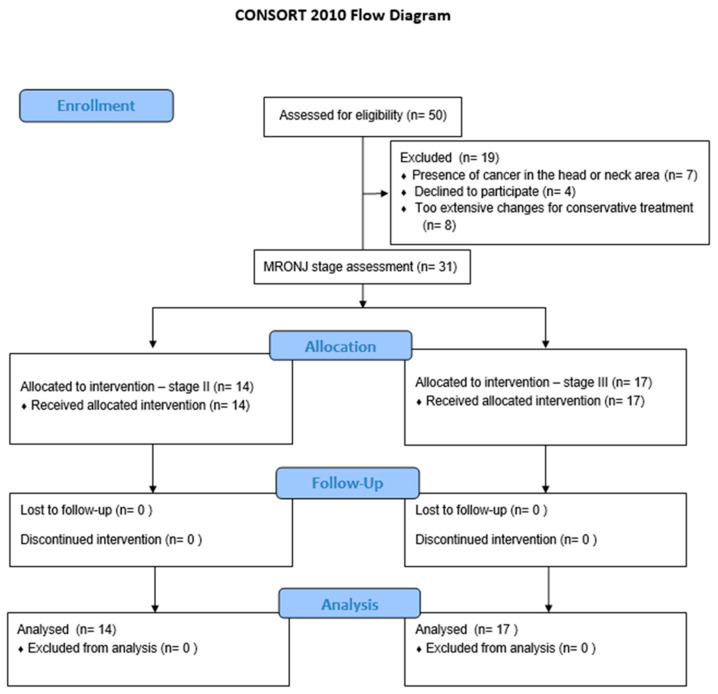
CONSORT Flow Diagram. Flow-chart showing inclusion, randomization, and participation throughout the study.

The sample size for this study was determined based on a power calculation to ensure sufficient statistical power to detect clinically meaningful differences in outcomes. Assuming a significance level (α) of 0.05 and a power (d) of 0.8, a minimum of 14 patients for each group was required to detect a medium effect size in the primary outcome measures. Due to the possibility of patients opting out of the study, the number of patients was increased to 31. This sample size was deemed adequate to ensure reliable analysis and valid conclusions regarding the effects of photobiomodulation therapy on MRONJ. Each patient underwent panoramic and CBCT scans prior to the implementation of the treatment regimen. The stage of MRONJ was assessed based on the classification established by the American Association of Oral and Maxillofacial Surgeons (AAOMS) in 2014 [46]. The group of 50 patients was examined (Figure 1), and only those with an initial diagnosis of MRONJ were included in the study. Inclusion criteria were limited to patients with a maximum Stage 3 MRONJ, restricted to half of the dental arch.

Patients were divided into two groups: Group 1 (G1, n = 14)—patients with stage 1 MRONJ, and Group 2 (G2, n = 17)—patients with stage 2 and 3 MRONJ. The STATISTICA package, version 13.3 (TIBCO Software Inc., Palo Alto, CA, USA), was used for statistical analysis of the study results. In the case of independent categorical data, the Pearson chi-square test was used, and in the case of paired binary variables, the McNemar test was used. The significance of differences in quantitative variables in the three stages of treatment was verified with the Friedman test. A *p* < 0.05 was considered statistically significant.

The group of 31 patients was examined and were included in the study with MRONJ. Inclusion criteria were limited to patients, with each stadium of MRONJ restricted to half of the dental arch. The study involved 21 women and 10 men aged between 44 and 86 years (mean M = 63, SD = 10 years). Stage 1 MRONJ was diagnosed in 14 individuals (45.2%) while 17 individuals had Stage 2 or 3 MRONJ. In total, 10 individuals had osteoporosis and 21 had cancer treatment. Patients were divided into two groups: Group 1 (G1, n = 14)—patients with Stage 1 MRONJ, and Group 2 (G2, n = 17)—patients with Stage 2 MRONJ.

**Figure 2:**
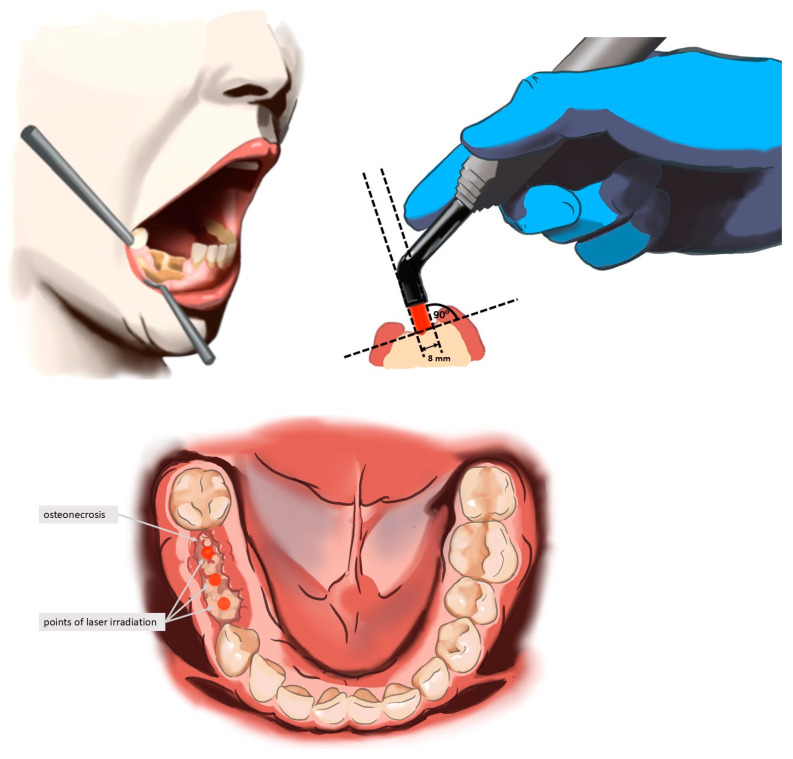
Methodology and points of laser irradiation according to clinical protocol.

The laser treatment protocol included the use of a diode laser (Lasotronix Smart M PRO, Piaseczno, Poland) for photobiomodulation (PBM) with the following parameters: 100 mW; continuous wave (CW); 635 nm wavelength; 4 J/cm^2^ energy density; applied for 20 s per point with spot and contact technique; irradiance for 1 point: 0.398 W/cm^2^; fluency for 1 point: 7.96 J/cm^2^, and for 4 points which was 1 appointment: 31.83 J/cm^2^; and tip diameter 8 mm (3 points from buccal surface, perpendicular for the lesion and 1 point on the floor of the mouth, total 80 s) during each session (see Figure 2). These parameters were selected based on prior evidence showing that low-level laser therapy using 635 nm wavelengths and an energy density around 4 J/cm^2^ effectively promotes fibroblast proliferation, collagen formation, angiogenesis, and pain reduction, which are essential mechanisms in the treatment of MRONJ [40,47]. Furthermore, these values fall within the well-established photobiomodulation therapeutic window, ensuring efficacy without thermal tissue damage. The protocol assumed 10 sessions at 3 days intervals. The laser was used with an 8 mm diameter tip at four points on the buccal surface, perpendicular to the outermost part of the lesion, as well as from the oral cavity surface. The treatment protocol consisted of 10 sessions at 3-day intervals. Three days prior to the initiation of PBM therapy, patients began antibiotic treatment with either amoxicillin and clavulanic acid (875 mg + 125 mg) or clindamycin (600 mg) every 12 h. Antibiotics were administered for a total of 14 days. During PBM sessions, all necessary biosafety measures were adhered to, including disinfection of the laser tip and the use of protective glasses by both the patient and the operator. Patients were instructed to rinse their mouth with 0.12% chlorhexidine 2 times daily for 20 s for 14 days starting on the first day of antibiotic therapy.

Pain levels were assessed using the Visual Analog Scale (VAS) before the treatment began and at 3 and 6 months after the start of therapy.

### Statistical Analysis

STATISTICA v. 13.3 (TIBCO Software Inc., Palo Alto, CA, USA), JASP (Version 0.19.3, JASP Team 2024) and Microsoft Excel spreadsheet were used to perform statistical analysis of clinical and survey results.

Due to the relatively rare incidence and highly specific inclusion criteria for medication-related osteonecrosis of the jaw (MRONJ), studies on MRONJ treatment commonly involve small patient cohorts. In this study, the smallest subgroup had 14 participants (stage I MRONJ). Given the limited patient pool, we performed a power analysis to assess the adequacy of our sample size. Assuming a medium-to-large effect size (Cohen’s d = 0.8), with an alpha level of 0.05, our sample size of n = 14 provided a statistical power of approximately 79%. According to standard methodological recommendations by Cohen [48], this power is adequate to detect clinically relevant differences.

Comparable studies on MRONJ treatment frequently employ similarly sized or even smaller samples, reflecting the inherent challenges in recruiting larger patient cohorts for such specialized research. For instance, recent MRONJ research has been published with similar small numbers of participants [49,50]. Such precedents highlight that despite the modest sample size, the current study aligns with the established standards and practices in the field.

Detailed statistical methodologies applied in this analysis are described below:

1. The assessment of the compliance of the empirical distributions of continuous quantitative variables (age and time to necrosis) and discrete variables (pain level on VAS scale) with theoretical normal distributions was performed using the Shapiro–Wilk test [51]. The critical significance level was *p* < 0.05.
2. For quantitative variables, mean values (M), standard deviations (SD), medians (Me), lower (Q1) and upper (Q3) quartiles, extreme values, and smallest (Min) and largest (Max) values were calculated. In tables and graphs, variables with a distribution close to normal were presented using the mean and standard deviation: M (SD), while variables with a distribution different from normal were presented as medians and quartiles: Me [Q1; Q3] [52].
3. For qualitative (nominal, e.g., sex, osteoporosis, and healing of the mucous membrane and gums) and ordinal (e.g., the degree of necrosis) variables, counts (n) and percentages (%) were calculated and collected in contingency tables [52].
4. Hypotheses about the lack of correlation between qualitative characteristics were verified using the Pearson chi-square test (χ^2^) [53,54] or Fisher’s exact test [55]. In the case of four-box tables (with dimensions of 2 × 2), the values of the odds ratio (OR) and their 95% confidence intervals (95% CI) were estimated.
5. The McNemar test [56,57] was used to analyze the differences in proportions in pairs of observations (in which each unit is assessed twice at 3 and 6 months after treatment).
6. The significance of differences in mean values in two groups for variables with close to normal distribution and homogeneous variances was tested using the Student’s *t*-test (*t*-test) [58,59].

## 3. Results

**Figure 3:**
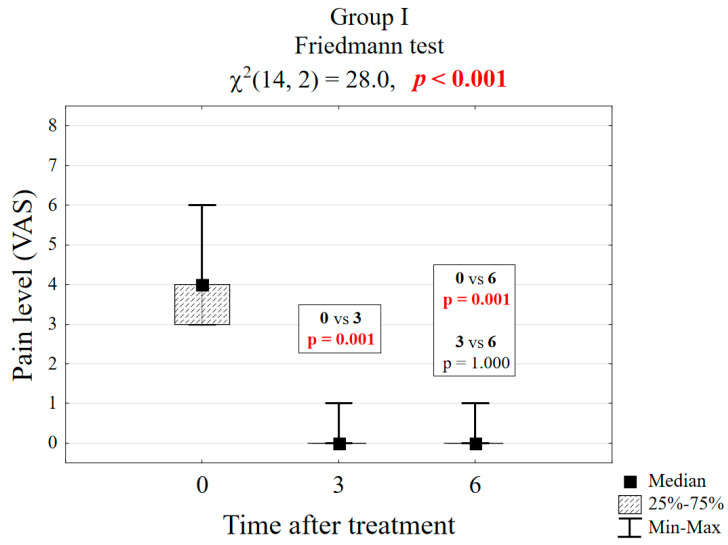
Box plot of pain reduction in group I.

**Figure 4:**
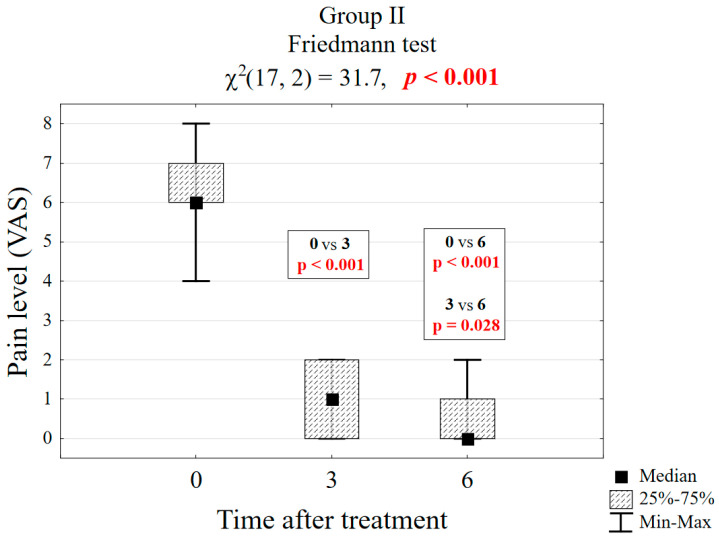
Box plot of pain reduction in group II.

Table 1 presents the results of pain reduction on the VAS scale for groups I and II. It is noticeable that in group I, the pain reduction after 3 months was significant at the level of *p* = 0.001 (4 vs. 0 score), and, after the next 3 months, it did not change significantly (observation could be ended after 3 months), see Figure 3. In group II, after 3 months (6 vs. 1 score), the difference was significant at the level of *p* < 0.001, but it progressed and only reached the level of 0 in the sixth month. It was advisable to continue the observation for a period of 6 months, see Figure 4.

The chance of pain reduction between 3 and 6 months after treatment in group II is twenty-six times greater compared to group I patients (see Table 2).

The result of the McNemar test (*p* = 0.855) suggests that there is no statistically significant difference between the compared proportions at the standard significance level of 0.05 (see Table 3). A 3-month follow-up period is sufficient.

**Figure 5:**
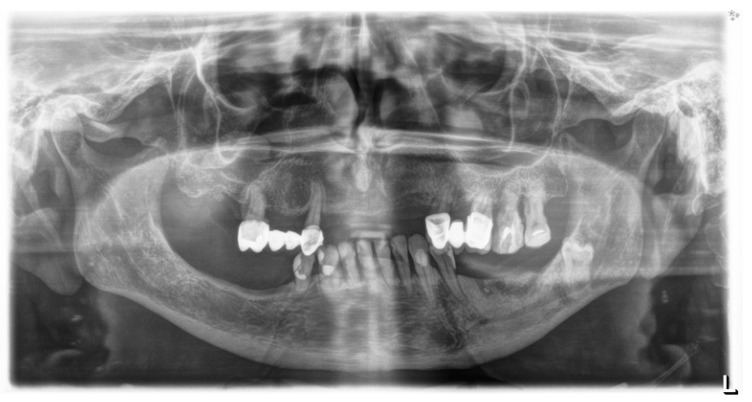
X-ray of the patient before treatment. Bone necrosis visible on the left side of the mandible.

**Figure 6:**
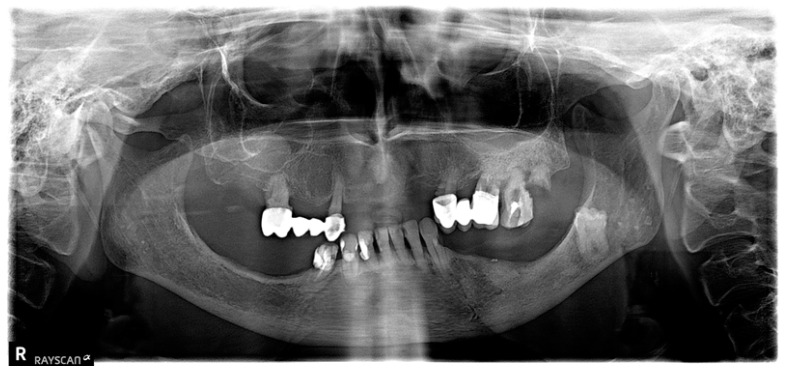
X-ray of the same patient as in Figure 5 after completion of treatment. Visible complete lack of necrotic changes in the mandibular bone.

After 3 months, all 31 patients showed improvement (one-grade reduction in necrosis). This change was the same in Group I as in Group II. After 6 months, all 14 patients in Group I showed improvement (one-grade reduction in necrosis). In Group II, improvement was also observed in all 17 patients, with one patient showing a two-grade reduction in necrosis, but this difference is not statistically significant (*p* > 0.05), see Table 4. Progress in treatment was also visible on X-rays. (see Figure 5 and Figure 6).

There is a statistically significant relationship between the degree of necrosis observed before treatment and the healing of the mucosa after treatment (Table 5). The chance of healing of the mucosa and gingiva in group I is eighty-seven times higher compared to patients in group II. In total, healing of the mucosa was observed in 100% of people in group I and 23.5% in group II. On average, for both groups, the use of PBM allows for healing of the mucosa in 58.1% of patients.

There is a statistically significant association between the degree of necrosis before treatment and the need for further treatment (*p* < 0.001), see Table 6. The analysis included 31 patients. In group 1, there was only one patient (6.2%) with degree I necrosis who required further treatment. In group II, 93.8% of patients with degree II or higher required further treatment despite the use of PBM therapy. The chance of complete recovery in group I patients is almost ninety-eight times higher compared to group II patients (OR = 97.5).

There was no significant connection between coexisting diseases and initial grade of the MRONJ. In the group of 21 women, no statistically significant association was observed between the initial degree of necrosis and the occurrence of osteoporosis (Table 7) or breast cancer (*p* > 0.05), see Table 8. And, in the group of 10 men, no statistically significant association was observed between the initial degree of necrosis and the occurrence of prostate cancer (*p* > 0.05), see Table 9.

## 4. Discussion

Medication-related osteonecrosis of the jaw (MRONJ) remains a significant challenge in clinical practice, with both surgical and nonsurgical approaches demonstrating variable efficacy [10,31,44,60]. Our study demonstrates the potential of photobiomodulation (PBM) therapy as a conservative treatment option, particularly for early-stage MRONJ. In patients with Stage 1 MRONJ, PBM achieved a 100% success rate, aligning with findings from previous research supporting the effectiveness of low-level laser therapy in promoting healing and reducing symptoms [31,43]. However, the response in stage II and III MRONJ was more limited. Although symptom relief was observed in all patients, the majority (88%) required further surgical intervention. The health status and underlying conditions of MRONJ patients frequently dictate treatment outcomes. For compromised patients, nonsurgical interventions like PBM may be preferable due to their lower invasiveness and reduced systemic impact [18,19]. Our findings are consistent with the literature, indicating that advanced disease stages and intravenous administration of antiresorptive drugs, such as bisphosphonates, are associated with poorer outcomes [11,61,62]. Furthermore, the duration of bisphosphonate therapy plays a critical role, with prolonged use (≥12 months) correlating with more severe disease and reduced treatment efficacy [63]. This highlights the importance of early detection and intervention to optimize outcomes.

The first working laser was presented by Theodore Maiman in 1960, and as early as 1965, Dr. Leon Goldman and Dr. Ralph Meyers began experimenting with its use in treating teeth and oral tissues. Since then, laser technology has been constantly developing and its use in dentistry is growing [64,65]. Today, hard lasers, such as CO_2_, Er:YAG, Er,Cr:YSGG, and Nd:YAG, are widely used in oral surgery, offering non-invasive treatment options and regenerative properties [66]. On the other hand, photobiomodulation (PBM) utilizes so-called cold lasers operating at low power [67,68]. PBM, through the biostimulation of tissues affected by MRONJ, provides numerous benefits. At the cellular level, it promotes the increased proliferation of fibroblasts and chondroblasts, enhances collagen synthesis, and stimulates osteogenesis. Additionally, PBM improves blood flow and stimulates the proliferation of endothelial cells. One of the most crucial benefits of non-invasive laser treatment for MRONJ is its significant analgesic effect, offering patients relief from pain [69,70,71].

Patients with Stage 2 and 3 MRONJ often endure significant pain due to ongoing inflammation, which severely impacts daily functioning, which may also occur in stage 0 MRONJ [72].

The meta-analysis from 2019 supports the positive effect of PBM on pain reduction, both as a monotherapy and as an adjunct to surgical treatment with biostimulation lasers [73]. Del Vecchio et al. (2022) [74] support the finding that photobiomodulation can play multiple roles in treatment strategies. Their review suggests that PBM offers pain relief and provides palliative care while also serving as a valuable adjunct to surgical interventions when used in a combined, multi-modal protocol. This integrated approach appears to enhance the healing and resolution of the pathological process, ultimately leading to a significant improvement in patients’ quality of life (QoL) [74]. In our study, patients across all MRONJ stages experienced notable pain relief after undergoing a laser therapy protocol comprising 10 sessions. This improvement was quantified using the VAS scale: patients with Stage 1 MRONJ showed a reduction in pain from a mean value of 3.85 to 0.07 while those with Stage 2 MRONJ experienced a decrease from 6.11 to 0.7 representing a reduction of 4.67 points. These findings align with the retrospective analysis conducted by Haviv et al. [72], which concluded that higher initial pain levels are associated with greater pain improvement following laser therapy. This trend was particularly evident in our Stage 2 MRONJ group.

Photobiomodulation can also be effectively applied as a preventive measure. Numerous studies have identified tooth extractions as a key trigger factor for MRONJ, underscoring the potential role of PBM in preventing complications in patients receiving antiresorptive drugs (ARDs) [25,74,75]. Primary prevention strategies should also consider the inflammatory condition of the tooth and surrounding tissues at the time of extraction [76]. Extractions performed due to periodontal inflammation, pulpitis, vertical root fractures, or periapical pathology significantly increase the risk of MRONJ development in patients on ARDs [77]. This highlights the importance of optimizing post-extraction healing and educating patients about the necessity of regular dental check-ups and examinations.

In a large clinical study on tooth extraction and MRONJ development, Vescovi et al. demonstrated the efficacy of PBM combined with antibiotic therapy in preventing osteonecrosis progression, achieving an MRONJ development rate of less than 1% after tooth extraction [42]. Comparatively, studies on patients undergoing bisphosphonate therapy without PBM intervention report significantly higher rates of MRONJ, further supporting the benefits of incorporating lasers into routine practice [78,79]. Additionally, while other preventive methods, such as hyperbaric oxygen therapy or plasma rich in growth factors (PRGF), have been explored, their effectiveness does not match the significant impact observed with low-power laser therapy in MRONJ prevention [80].

The current body of literature highlights significant progress in the use of PBM for MRONJ treatment, yet there are gaps that merit further exploration. Studies such as those by Vescovi et al. [42], El Mobadder et al. [43], or others emphasize the benefits of combining PBM with antibiotics or minimally invasive laser surgical interventions. However, standardized protocols for laser parameters and session frequency remain undefined. Additionally, PBM’s role in modifying systemic factors, such as reducing overall inflammation or supporting bone turnover at the molecular level, warrants further investigation [37,40].

A limitation of this study is the relatively small number of participants, which, despite being common in MRONJ studies due to stringent inclusion criteria, limits the statistical power and generalizability of the results obtained. Additionally, the absence of a control group without PBM treatment restricts our ability to conclusively differentiate the effects of laser therapy from those potentially achieved by antibiotic treatment alone or spontaneous healing. Further studies with larger sample sizes and controlled designs are required to substantiate these preliminary findings.

The average age of patients in our study was approximately 66 years for women and 56 years for men, aligning with median findings from recent research published in 2023 [61]. Advanced age often presents a barrier to performing comprehensive surgical procedures due to patients’ medical history and underlying conditions, which are frequently associated with malignancies [81,82].

The findings of our study, supported by similar research [72,73], raise the question of whether PBM could be integrated into early intervention strategies for patients identified as high-risk due to prolonged ARD use or intravenous drug administration. A randomized controlled trial comparing PBM with other emerging treatments, such as PRGF or hyperbaric oxygen therapy, could provide clarity on its relative efficacy [61,80,81,82]. Finally, given the promising pain relief observed in Stage 2 patients, exploring PBM’s impact on quality-of-life metrics, such as eating, speaking, and social functioning, could further expand its role in MRONJ management.

## 5. Conclusions

This study partially supports the thesis that non-invasive laser biostimulation can serve as an effective alternative to surgery, particularly in early-stage MRONJ. Laser treatment demonstrated complete healing in patients with Stage 1 MRONJ, while also promoting the regression of inflammation and significant pain reduction regardless of the disease’s stage. Additionally, PBM contributed to slowing the progression of bone necrosis. However, in very advanced cases, surgical intervention remains the most effective treatment option [83,84].

Given the dynamic nature of MRONJ treatment guidelines and the rising incidence of malignancies, which may lead to an increase in MRONJ cases, there is a growing responsibility for oncologists and surgeons to educate patients on the potential risks. This is especially critical when initiating ARD therapy, as early awareness and preventive measures can significantly reduce the likelihood and severity of MRONJ [85].

Our study confirms the effectiveness of non-invasive laser biostimulation for early-stage MRONJ, demonstrating complete healing in stage I cases and significant improvements in inflammation and pain across all stages. While advanced cases still require surgical intervention for optimal outcomes, PBM remains a valuable adjunctive treatment, particularly for patients unable to undergo surgery. These findings emphasize the need for continued research into standardized PBM protocols and its integration into MRONJ treatment and prevention strategies.

## Figures and Tables

**Table 1:** Pain reduction in groups I and II.

Variable	Group I n = 14	Group II n = 17	*p*-Value
Pain level before treatment on VAS scale			<0.001 ^d^
M ± SD	3.9 ± 0.9	6.1 ± 1.0	
Me [Q1; Q3]	4 [3; 4]	6 [6; 7]	
Min–Max	3–6	4–8	
Pain level 3 months after treatment on VAS scale			0.004 ^d^
M ± SD	0.1 ± 0.3	1.1 ± 0.9	
Me [Q1; Q3]	0 [0; 0]	1 [0; 2]	
Min–Max	0–1	0–2	
Pain level 6 months after treatment on VAS scale			0.067 ^d^
M ± SD	0.1 ± 0.3	0.5 ± 0.8	
Me [Q1; Q3]	0 [0; 0]	0 [0; 1]	
Min–Max	0–1	0–2	

^d^ *p*-value Student’s *t*-test.

**Table 2:** Evaluation of the chances of pain reduction between 3 and 6 months.

	Pain Reduction Between 3 and 6 Months of Treatment		Test Results	OR [95% CI]
	Yes	No		
Group II	8 (47.1)	9 (52.9)	*p* = 0.003 ^b^	25.9 [1.33; 504]
Group I	0 (0.0)	14 (100.0)		1.00 (Ref.)

^b^—Fisher’s exact test, OR—odds ratio.

**Table 3:** McNemar’s test for MRONJ healing at 3 and 6 months.

Healed After 6 Months	Healed After 3 Months		Chi-Square McNemar’s A/D
	Yes (0°) n = 14	No (I° or II°) n = 17	
Yes (0°) n = 15	14 (45.2%)	1 (3.2%)	*p* = 0.855 ^e^
No (I° or II°) n = 16	0 (0.0%)	16 (51.6%)	

^e^—McNemar test.

**Table 4:** Quantitative assessment of the reduction in the degree of MORNJ.

	Group I	Group II	Test Result
Improved by one degree, n (%)	14 (100.0)	16 (94.1)	*p* = 1.000 ^b^
Improved by two degrees, n (%)	0 (0.0)	1 (5.9)	

^b^—Fisher’s exact test.

**Table 5:** Assessment of mucosal healing.

The Degree of Necrosis	Healing of the Mucous Membrane and Gums		Test Results	OR [95% CI]
	Yes (n = 18)	No (n = 13)		
I, n (%)	14 (77.8)	0 (0.0)	*p* < 0.001 ^b^	87.0 [4.27; 1773]
II and III, n (%)	4 (22.2)	13 (100.0)		1.00 (Ref.)

^b^—Fisher’s exact test, OR—odds ratio.

**Table 6:** Need for further treatment.

	Need for Further Treatment		Test Results	OR [95% CI]
	No (n = 15)	Yes (n = 16)		
Group I	13 (86.7)	1 (6.2)	*p* < 0.001 ^b^	97.5 [7.90; 1203]
Group II	2 (13.3)	15 (93.8)		1.00 (Ref.)

^b^—Fisher’s exact test, OR—odds ratio.

**Table 7:** Correlation between MRONJ grade and osteoporosis incidence.

Initial Grade of Necrosis	Osteoporosis		*p*
	Yes (n = 9)	No (n = 12)	
I (n = 9)	5 (55.6%)	4 (33.3%)	0.465 ^c^
II (n = 10)	4 (44.4%)	6 (50.0%)	
III (n = 2)	0 (0.0%)	2 (16.7%)	

^c^—Chi-square Pearson test.

**Table 8:** Correlation between MRNJ grade and breast cancer incidence.

Initial Grade of Necrosis	Breast Cancer		*p*
	Yes (n = 9)	No (n = 12)	
I (n = 9)	3 (33.3%)	6 (50.0%)	0.820 ^c^
II (n = 10)	5 (55.6%)	5 (41.7%)	
III (n = 2)	1 (11.1%)	1 (8.3%)	

^c^—Chi-square Pearson test.

**Table 9:** Correlation between MRNJ grade and prostate cancer incidence.

Initial Grade of Necrosis	Prostate Cancer		*p*
	Yes (n = 6)	No (n = 4)	
I (n = 5)	2 (33.3%)	3 (75.0%)	0.714 ^c^
II (n = 4)	3 (50.0%)	1 (25.0%)	
III (n = 1)	1 (16.7%)	0 (0.0%)	

^c^—Chi-square Pearson test.

## Data Availability

The original contributions presented in this study are included in the article. Further inquiries can be directed to the corresponding author.

## Abbreviations

ARDs	Antiresorptive Drugs
CBCT	Cone-Beam Computed Tomography
CW	Continuous Wave
Er:YAG	Erbium-Doped Yttrium Aluminum Garnet
J/cm^2^	Joules per Square Centimeter
MRONJ	Medication-Related Osteonecrosis of the Jaw
mW	Milliwatt
nm	Nanometer
OR	Odds Ratio
PBM	Photobiomodulation
PRGF	Plasma Rich in Growth Factors
QoL	Quality of Life
SD	Standard Deviation
VAS	Visual Analog Scale
W/cm^2^	Watts per Square Centimeter
